# Mangiferin promotes osteogenic differentiation and alleviates osteoporosis in the ovariectomized mouse *via* the AXL/ERK5 pathway

**DOI:** 10.3389/fphar.2022.1028932

**Published:** 2022-10-28

**Authors:** Jinwen He, Xingwen Wang, Dacheng Zhao, Bin Geng, Yayi Xia

**Affiliations:** Department of Orthopaedics, Orthopaedics Clinical Medicine Research Center of Gansu Province, Intelligent Orthopedics Industry Technology Center of Gansu Province, Lanzhou University Second Hospital, Lanzhou, Gansu, China

**Keywords:** osteoporosis, osteogenic-differentiation, ovariectomized, mangiferin, Axl, Erk5

## Abstract

Mangiferin is a xanthone glucoside extracted from multiple plants, which has been shown to inhibit bone resorption and alleviate osteoporosis. However, the effect of purified Mangiferin on osteoporosis and its specific mechanisms is unknown. This study aimed to explore whether Mangiferin can promote osteogenic differentiation and alleviate osteoporosis in ovariectomized (OVX) mice and explore the potential mechanisms. Different concentrations and durations of Mangiferin were applied to MC3T3-E1 cells. The optimal concentration and duration of Mangiferin were determined by evaluating the cell viability *via* cell count kit-8 (CCK-8). The gene and protein expressions of AXL, ERK5, and osteogenic differentiation markers, including BMP2, Collagen1, OPN, Osterix, and Runx2, were detected using western blotting, qRT-PCR, immunofluorescence, and flow cytometry. Mangiferin was administered to OVX mice, and the severity of osteoporosis was evaluated by H and E staining, immunohistochemistry (IHC), microscopic computed-tomography (micro-CT) scanning, western blotting, and immunofluorescence of bone tissue. We found that Mangiferin promoted osteogenic differentiation in a dose-dependent manner at concentrations less than 30 μM. The 30 μM Mangiferin significantly upregulated the expression of AXL, ERK5, and osteogenic differentiation, including the ALP activity, percentage of alizarin red, and the levels of osteogenic differentiation markers. However, these expression levels decreased when AXL was knocked down in MC3T3-E1 cells and it could not be rescued by Mangiferin. Mangiferin relieved osteoporosis in OVX mice without causing severe organ damage. This study concluded that Mangiferin promoted osteogenic differentiation of MC3T3-E1 cells and alleviated osteoporosis in OVX mice. The potential mechanism was *via* the AXL/ERK5 pathway.

## Introduction

The incidence of osteoporosis has been rising yearly with the aging population (Compston et al., 2019; Anam and Insogna, 2021). Postmenopausal osteoporosis is the most prevalent form affecting the quality of life of older women, resulting in osteoporotic fractures, bedding-related complications, and loss of morbidities (Jackson and Mysiw, 2014; Watts et al., 2021). The pathogenesis of osteoporosis has been linked to the functions of osteoblasts and osteoclasts, which have opposing roles in disease progression. Osteoporosis is caused by the disruption of the balance between the two cell types, especially in elderly patients. Current treatment options for osteoporosis rely heavily on inhibiting the function of osteoclasts, which reduces bone loss and absorption. However, osteoblast function has not been thoroughly investigated. Only a few drugs targeting the osteoblast have been developed.

**FIGURE 1:**
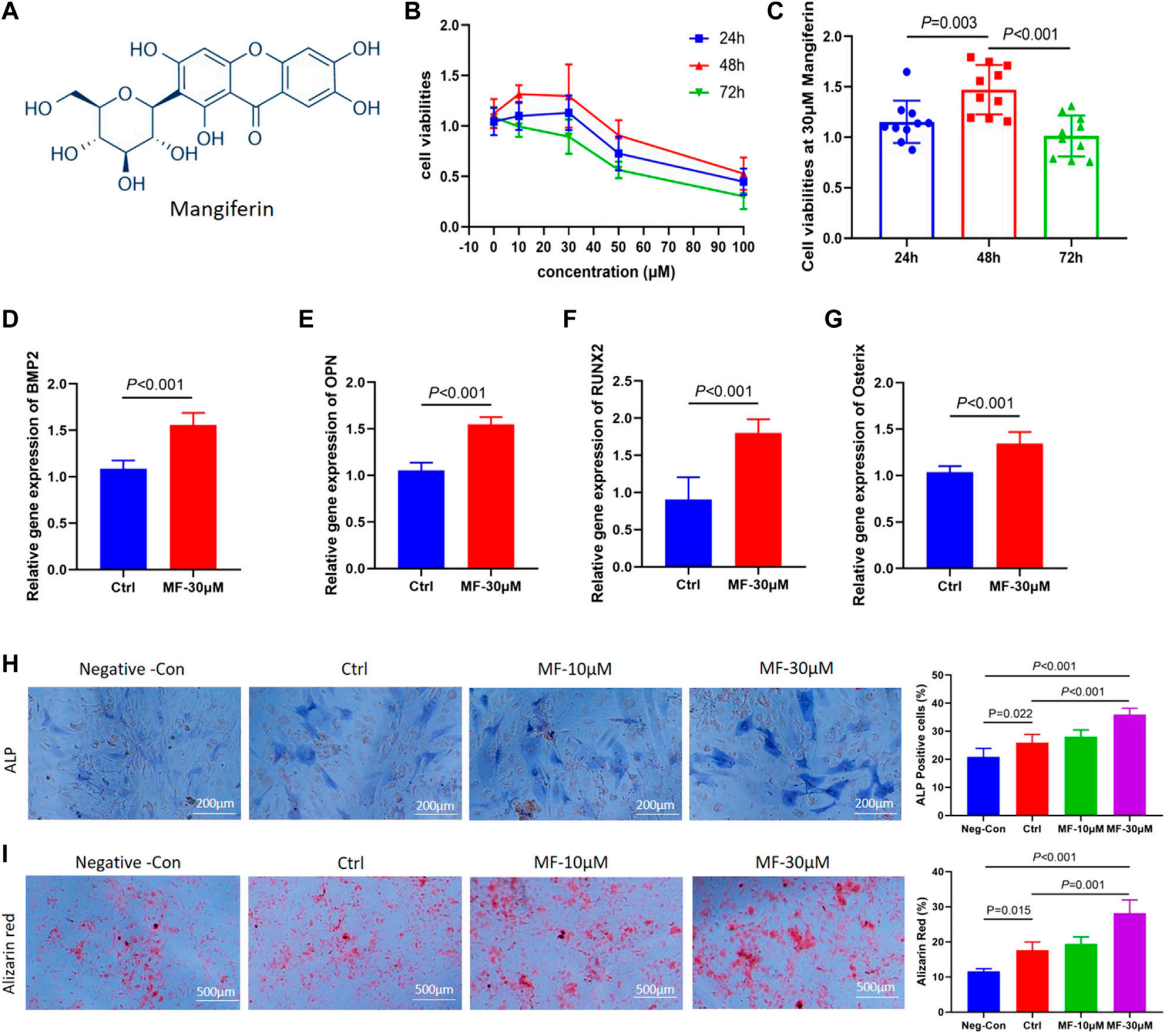
**(A)**. The molecular formula of Mangiferin. **(B)** Line chart view. The cell viability of MC3T3-E1 cells decreased when the concentration of mangiferin exceed 30 μM, when the intervention duration was 48 h, the cell viability presented the highest level. **(C)** Histogram view. When compared with 24 h and 72 h, the cell viability of MC3T3-E1 cells significantly increased when the duration was 48 h (*p* < 0.01). **(D–G)**. The relative gene expression of BMP2, OPN, Osterix, and Runx2 significantly increased when the MC3T3-E1 cells were intervened by MF-30 μM mangiferin in the osteogenic culture for 14 days (compared with the control group, *p* < 0.01, respectively). **(H)** Microscopic observation. The ALP activity in the MF-10 μM and MF-30 μM significantly increased when compared with the control group (*p* < 0.05, respectively). **(I)** In visual observation, the Alizarin intensity red in the 10 μM and 30 μM significantly increased when compared with the control group (*p* < 0.05, respectively). There was no significant difference in the percent of Alizarin red between MF-30 μM and the positive-control group (*p* > 0.05). Note: MF, Mangiferin. Neg-Con, Negative control. Ctrl, osteogenic induction group.

Mangiferin (MF) is a xanthone glucoside extracted from multiple plants, including *Mangifera indica* Linn (Family: Anacardiaceae, Genus: Mangifera, Figure 1A) (Muruganandan et al., 2005; Gold-Smith et al., 2016; Morozkina et al., 2021). It has been reported to have anti-inflammatory, antioxidant, anticancer, anti-diabetic, cardiovascular, and nervous system effects (Gold-Smith et al., 2016; Hou et al., 2018; Sadhukhan et al., 2018; Li et al., 2020; Morozkina et al., 2021). According to traditional Chinese medicine, Mangiferin has been a bioactive compound of the traditional Chinese medicine Er-Xian Decoction, which has been used for osteoporosis treatment (Qin et al., 2008). Li et al. (1998) found that Mangiferin has been an active constituent of Tsu-kan-gan, which has been used for inhibiting *in vivo* and *in vitro* bone resorption. As a flavonoid, Mangiferin is also extracted from the Anemarrhena Asphodeloides Bunge and found to have anti-osteoporosis properties (Wang et al., 2014). Ang et al. (2011) found that Mangiferin was able to attenuate osteoclastogenesis and bone resorption by activating the nuclear factor-k-gene binding (NF-κB) and extracellular regulated protein kinase (ERK) pathway. According to Sekiguchi et al. (2017), Mangiferin enhances osteoblast differentiation and suppresses osteoclast differentiation.

AXL is a member of the receptor tyrosine kinase TAM family, current studies have found that the only ligand that binds to the AXL protein molecule is growth arrest-specific 6 (Gas6), and the homodimer generated by ligand binding activates AXL (which is the most typical of Axl). It has been proven that Gas6/AXL signaling regulates cell survival and proliferation through the MAPK/ERK pathway (Li et al., 2019a). However, the effect of the AXL/ERK signaling pathway on the proliferation and differentiation of osteoblasts remains unknown.

Thus, Mangiferin is an active compound in traditional Chinese medicine that can alleviate osteoporosis. The purified Mangiferin inhibits osteoclastogenesis and bone resorption, indicating that it can be a promising osteoporosis treatment. However, most of the published studies on the effects of Mangiferin in traditional Chinese medicine focused on osteoclast function and bone resorption. Furthermore, there is minimal evidence of the impact of purified Mangiferin on osteoblasts, and the precise mechanism is unknown.

This study aimed to investigate the effect of purified Mangiferin on osteoporosis and to explore the processes behind Mangiferin’s effect on osteoblastic function.

## Materials and methods

### Evaluation of mangiferin cytotoxicity and osteogenic potential *in vitro* using MC3T3-E1 pre-osteoblasts

The MC3T3-E1 cells (the third passage number) were purchased from Xiehe University (Beijing, China). Cells were cultured in the alpha minimum essential medium (α-MEM) (with 10% fetal bovine serum, Gibco, Melbourne, Australia), with 5% carbon dioxide (CO_2_) at 37°C. Mangiferin was purchased from Selleck (Selleck Chemicals LLC, Houston, United States). For cell differentiation, the MC3T3-E1 cells were cultured in osteogenic culture (10 mM β-glycerophosphate, 50 µM ascorbic acid, 1 µM dexamethasone) supplemented with optimal concentrations of Mangiferin. The total intervention duration was 14~21 days, and the culture was replaced every 3 days.

To explore the best concentration and intervention time for Mangiferin, cell viability was evaluated using the cell count kit-8 (CCK-8). Briefly, cells were planted in the 96-well plates with a density of 3,000 cells per well. After culturing for 24 h, cells were treated with 10, 20, 30, 50, and 100 µM of Mangiferin for 24, 48, and 72 h, respectively. After that, the CCK-8 kit and culture were added to the wells with a 1:10 ratio, and the plates were incubated at 37°C for 2 h, the optical density (OD) was measured using a Microplate reader (Biotek, Vermont, United States). The cell viabilities were calculated *via* the following formula: cell viabilities = (OD treatment-OD blank)/(OD control-OD blank).

### Construction of AXL knockdown cells by using lentiviral transfection

AXL was knocked down in the MC3T3-E1 cell using the AXL^−/−^ Lentivirus (Genechem, Shanghai, China). The optimal Multiplicity of Infection (MOI) was 50, which was confirmed by Negative Control Lentivirus (sh-NC). The optimal concentration of Puromycin was 7 μg/ml, which was confirmed in MC3T3-E1 cells. AXL^−/−^ Lentivirus (sh-AXL) was transfected to the MC3T3-E1 cells using one-half volume transfection. It was compulsory to achieve more than 70% efficiency of the AXL gene knockdown, which was verified through qRT PCR and western blotting.

### Establishment of the ovariectomized model and intervention

This study was approved by the animal ethics committee (D2020-37) and strictly followed animal welfare standards. SPF female C57BL/6 mice, aged six to 8 weeks, weighed 20–30 g, were purchased from Lanzhou Institute of Veterinary Medicine, Chinese Academy of Sciences. The ovariectomies (OVX) were performed using an aseptic technique to stimulate postmenopausal osteoporosis. In detail, mice were anesthetized with 1% pentobarbital sodium (50 mg/kg), and bilateral ovariectomies were performed in the OVX group. In the SHAM group, the ovaries were exposed but were not resected. After the incision was sutured, mice were put on a heating pad to keep warm, and normal saline was given by intraperitoneal injection to prevent dehydration until recovery from anesthesia. After surgery, mice were bred in the Specific Pathogen Free (SPF) animal breeding center, in constant temperature and humidity, with alternation of light and darkness, free of administration of food and water.

Mangiferin was dissolved in the dimethyl sulfoxide (DMSO) and cosolvent (including PEG300, Tween 80, and ddH_2_O) according to the manufacturer’s instructions. On the third day after OVX surgery, mice were randomly divided into four groups: OVX + Mangiferin group (OVX mice intraperitoneally injected with 40 mg/kg/d Mangiferin) (Huang et al., 2020). OVX + DMSO group (OVX mice intraperitoneally injected with the same dose of DMSO and solvent), OVX group (OVX mice intraperitoneally injected by the same dose of 0.9% normal saline), and SHAM group (SHAM mice intraperitoneally injected by the same dose of 0.9% normal saline). The Mangiferin/DMSO/normal saline was given quaque die (qd), and the course of treatment lasted for 8 weeks.

### Tissue collection

At the end of the treatment, mice were anesthetized with 1% pentobarbital sodium (50 mg/kg). To assess organ toxicity, the heart, liver, spleen, lung, and kidneys were resected and fixed in 4% paraformaldehyde for H and E. The bilateral femurs were resected and fixed in 4% paraformaldehyde, decalcified to perform H and E staining, immunohistochemistry (IHC) analysis, and immunofluorescence. For micro-CT analysis, the femurs were preserved in 70% ethanol. For western blotting and qRT-PCR analysis, the femurs were quickly frozen in liquid nitrogen and stored at −80°C.

### qRT-PCR

The osteogenic differentiation biomarkers, include BMP2, OPN, Osterix, and Runx2. The signaling pathway biomarkers including Gas6, AXL, and ERK5 were measured by qRT-PCR. The primer sequences are listed in Table 1. Total RNA was extracted using Trizol (Accurate Biology, Changsha, China). Trizol was added to the cell flasks for 7 minutes. For bone tissues, the femur was ground with Trizol in liquid nitrogen. The crushed tissues were collected in a tube, and RNA was extracted by chloroform, purified with isopropyl alcohol, and washed with ethanol. The concentration of total RNA was determined using Nanodrop 2000, and the RNA was then reverse transcribed and amplified according to the instructions (Takara, Shiga, Japan). The reverse transcription was performed using the GE4852T (Bio-Gener Technology, Hangzhou, China), and the amplification was performed by the LightCycler 96 (Roche Diagnostics, Basel, Switzerland).

**TABLE 1:** Primers’ sequence of qRT-PCR.

Gene name	Sequence
RUNX2	F: CCT​TCA​AGG​TTG​TAG​CCC​TC
	R: GGA​GTA​GTT​CTC​ATC​ATT​CCC​G
Osterix	F: GAT​GGC​GTC​CTC​TCT​GCT​TG
	R: GGG​CTG​AAA​GGT​CAG​CGT​AT
OPN	F: AAA​CAC​ACA​GAC​TTG​AGC​ATT​C
	R: TTA​GGG​TCT​AGG​ACT​AGC​TTG​T
BMP2	F: AGT​AGT​TTC​CAG​CAC​CGA​ATT​A
	R: CAC​TAA​CCT​GGT​GTC​CAA​TAG​T
Gas6	F: CAT​CTT​ACC​GTG​TGT​GCC​CT
	R: GAC​GAT​CCA​GGT​GCT​ATC​CG
AXL	F: GGG​GAT​TAC​TAC​CGC​CAA​GG
	R: TCT​CCC​ACA​TTG​TCA​CAC​CG
ERK5	F: ATC​CTC​AAA​CAC​TTC​AAA​CAC​G
	R: GAT​CTG​AAT​TCT​CCA​TAG​GGC​A

### Alkaline phosphatase staining and alizarin red staining

Alkaline phosphatase (ALP) staining and alizarin red staining were used to evaluate osteogenic differentiation. Cells were planted in the 6-well plates with a density of 5 × 10^3^ cells per well. In the Negative control (Neg-Con) group, the MC3T3-E1 cells were cultured with 10% fetal bovine serum. In the osteogenic induction (Ctrl) group, cells were cultured in the osteogenic culture. In the mangiferin group, after culturing for 24 h, osteogenic culture was supplemented with 10, 20, and 30 µM of Mangiferin, the culture and Mangiferin were refreshed every 3 days. For alkaline phosphatase staining (Solarbio, Beijing, China), cells were cultured for 14 days, cleaned with PBS solution, fixed by ALP fixative solution for 15 min, cleaned with ddH2O, and stained with ALP Incubation solution (B1AS-BI staining solution: B2 FBB staining solution = 1:1) for 20 min, cleaned with ddH2O, stained in the Nuclear Solid Red solution for 5 min, cleaned with PBS solution, the images were captured under the microscope. For alizarin red staining, cells were cultured for 21 days, cleaned with PBS solution, fixed with 4% Paraformaldehyde for 15 min, cleaned with ddH2O, stained with alizarin red staining solution (1%, pH 4.2, Solarbio, Beijing, China) for 30 min, cleaned with ddH2O, images were captured using the camera and microscopy, respectively.

### Immunofluorescence

The cells were planted in the coverslips with a density of 5 × 10^3^ cells per slip and cultured in 6-well plates. When the degree of fusion was achieved to 30%, the cells were treated with 10, 20, and 30 µM of Mangiferin, respectively. For immunofluorescence, the cells were washed with PBS solution, fixed in 4% paraformaldehyde, rendered transparent with the 0.1% triton, and blocked with 10% goat-anti-rabbit serum. Then were incubated with primary antibodies, including anti-AXL (1:200, Abcam, Cambridge, United Kingdom), anti-ERK5 (1:300, Abcam, Cambridge, United Kingdom), anti-OPN (1:300, Abcam, Cambridge, United Kingdom), and anti-Collagen1 (1:300, Abcam, Cambridge, United Kingdom). The cells were then incubated with the 4,6-diamino-2-phenyl indole (DAPI) and secondary antibodies, including Alexa Fluor 488 luciferin and Alexa Fluor 594 luciferin, in the dark. According to the instructions, the mitochondrial membrane potential (MMP) was stained using the mitochondrial membrane potential fluorescent probes (AAT Bioquest, California, United States). Three images from the slides were acquired using the fluorescence microscope (Olympus, Japan), and the expression level of AXL, ERK5, OPN, and Collagen1 were evaluated by measuring the mean immunofluorescence intensity, which was calculated by integrated optical density (IOD)/positive pixel area (Dogan et al., 2019; Liu et al., 2020).

### Western blotting

The cells were planted in the culture dish, and when the degree of fusion was achieved to 30%, the cells were treated with 10, 20, and 30 µM of Mangiferin for 14 days. PBS solution was used to wash the cells and treated with radio-immunoprecipitation assay (RIPA) and phenylmethanesulfonyl fluoride (PMSF) (100:1, Beyotime Biotechnology, Shanghai, China). For bone tissues, the bone was washed with PBS solution, ground in liquid nitrogen, and treated with RIPA and PMSF (100:1). The lysis solution was collected in the tube and centrifuged at 12,000 rpm for 20 min at 4°C. The supernatant was pipetted into a new tube and quantified by a bicinchoninic acid (BCA) kit (Solarbio, Beijing, China). It was diluted in loading buffer, boiled at 100°C for 10 min, and then stored at −20°C for analysis. Western blotting was performed on proteins. Briefly, the proteins were separated by sodium dodecyl sulfate-polyacrylamide gel electrophoresis (SDS-PAGE), transferred to a polyvinylidene fluoride (PVDF) membrane (Millipore Sigma, Burlington, MA), blocked with a blocking reagent, and incubated with primary antibodies at 4°C overnight; including anti-Gas6 (1:1000, Abcam, Cambridge, United Kingdom), anti-AXL (1:1000, Abcam, Cambridge, United Kingdom), anti-ERK5 (1:1000, Cell Signaling Technology, Massachusetts, United States), anti-phospho ERK5 (1:1000, Cell Signaling Technology, Massachusetts, United States), anti-OPN (1:1250, Abcam, Cambridge, United Kingdom), anti-Osterix (1:1250, Abcam, Cambridge, United Kingdom), and anti-Runx2 (1:1250, Abcam, Cambridge, United Kingdom). On the second day, the proteins were incubated with anti-rabbit or anti-mouse antibodies. The relative protein expression was quantified by comparing it with glyceraldehyde-3-phosphate dehydrogenase (GAPDH) (Affinity, Melbourne, Australia). The incubated PVDF membrane was exposed to enhanced chemiluminescence (ECL) using a chemiluminescence imaging system (MiniChemi, Beijing, China), and the mean gray value of each sample was measured by using the ImageJ software (National Institutes of Health, Maryland, United States). The expression of OPN, RUNX2, Osterix, Gas6, and AXL was quantified by comparison with that of GAPDH. The expression of phosphorylated-ERK5 was quantified by comparison with that of ERK5.

### Flow cytometry

Cells were cultured in the 6-well plates. When the degree of fusion reached 20%–30%, cells were treated with different concentrations of Mangiferin (dissolved in DMSO) for the optimal duration, and the same dose of DMSO was added to the DMSO group. After incubation for 48 h, the cells were collected and stained by cell cycling staining kits (Multisciences, Hangzhou, China) and Annexin V-FITC/PI cell apoptosis detection kit (Abcam, Cambridge, United Kingdom), according to the manufacturer’s instructions. The flow cytometer (Beckman Coulter, Brea, CA, United States) detected the cell cycle and apoptosis using specific antibodies. The percentage of cell cycling and apoptosis was calculated, and a comparison between groups was performed. The reactive oxygen species (ROS) level was measured using the DCFH-DA Reactive Oxygen Species Assay Kit (MedChemExpress LLC, New Jersey, United States), according to the manufacturer’s instructions. The results were analyzed using FlowJo software (FlowJo™, Ashland, United States).

### H and E staining

All mice were treated for eight consecutive weeks, after that, tissues were collected, the specific procedures can be seen in the supplementary materials. For H and E, the organs and femur were fixed with 4% paraformaldehyde, dehydrated, embedded in paraffin, and cut into 4 µm thick slices. The slices underwent gradient dehydration and rehydration and were then stained by H and E. The toxicity of Mangiferin was evaluated by observing the morphology of organs and the presence of inflammatory cell infiltration. The osteoporosis of the femur was assessed by observing the morphology and layout of trabecular bone.

### Immunohistochemistry

The slides underwent dewaxing, dehydration, rehydration, antigen retrieval, and blocking. They were then incubated with primary antibodies overnight, including anti-OPN (1:300, Abcam, Cambridge, United Kingdom), anti-RUNX2 (1:300, Abcam, Cambridge, United Kingdom), and anti-ERK5 (1:300, Affinity, Melbourne, Australia), followed by incubation with secondary antibodies (anti-mouse and anti-rabbit; Servicebio, Wuhan, China). The images were captured by the microscope system (Olympus, Japan); in each slice, three images from different visions were captured. The experiment was repeated three times. The H-score was calculated for expression analysis of OPN, Runx2, and ERK5, using the following formula: H-Score = ∑(pi×i) = (percentage of weak intensity ×1) + (percentage of moderate intensity ×2) + (percentage of strong intensity ×3) (Maclean et al., 2020).

### Micro-computed tomography

The femurs were scanned by the three-dimensional Micro-Computed Tomography (Micro-CT) system. In addition, a 3D reconstruction was performed. To determine the severity of osteoporosis, the bone mineral density (BMD), bone volume (BV), bone surface (BS), total volume (TV), bone volume fraction (BV/TV), bone surface density (BS/TV), trabecular separation/spacing (Tb. Sp), trabecular thickness (Tb. Th), trabecular number (Tb. N), and connectivity (Conn) were measured and analyzed.

### Statistical analysis

SPSS 24.0 (SPSS Inc., Chicago, United States) was used for statistical analysis. The Kolmogorov-Smirnov test analyzed the normality of data. Comparison between two normally distributed quantitative data was performed using the independent *t*-test, and the comparison among multiple samples was performed using the one-way analysis of variance (ANOVA). The Mann-Whitney U and Kruskal Wallis H tests were used if the data were not normally distributed. Qualitative data were presented as percentages, and the Chi-square test was used. *p* < 0.05 was considered statistically significant. All experiments were repeated three times.

## Results

### Mangiferin modulates MC3T3-E1 viability in a dose- and time-dependent manner

Among 0, 10, 30, 50, and 100 µM concentrations, the cell viabilities presented an increasing trend from 10 to 30 μM, while it presented a decreasing trend above 30 μM (Figure 1B). Thus, the 30 µM concentration was chosen to determine the optimal concentration of Mangiferin intervention. At 30 µM concentration, the cell viability presented the highest level when treated for 48 h (compared with 24 and 72 h, *p* < 0.01, Figure 1C), indicating that a concentration less than 30 µM and a duration of 48 h significantly promoted MC3T3-E1 cell proliferation.

### Mangiferin promoted osteogenic differentiation of MC3T3-E1 cells

The qRT-PCR demonstrated that when the concentration of Mangiferin was 30 μM, the gene expression of BMP2, OPN, Osterix, and RUNX2 significantly increased compared to the control group (*p* < 0.01, Figures 1D–G). The ALP and Alizarin red staining observed under the microscope demonstrated that 10 and 30 µM Mangiferin significantly promoted osteogenic differentiation. There was a rising trend of ALP and Alizarin red activity when the concentration was increased from 10 to 30 µM compared to the control group (*p* < 0.05, Figures 1H,I).

**FIGURE 2:**
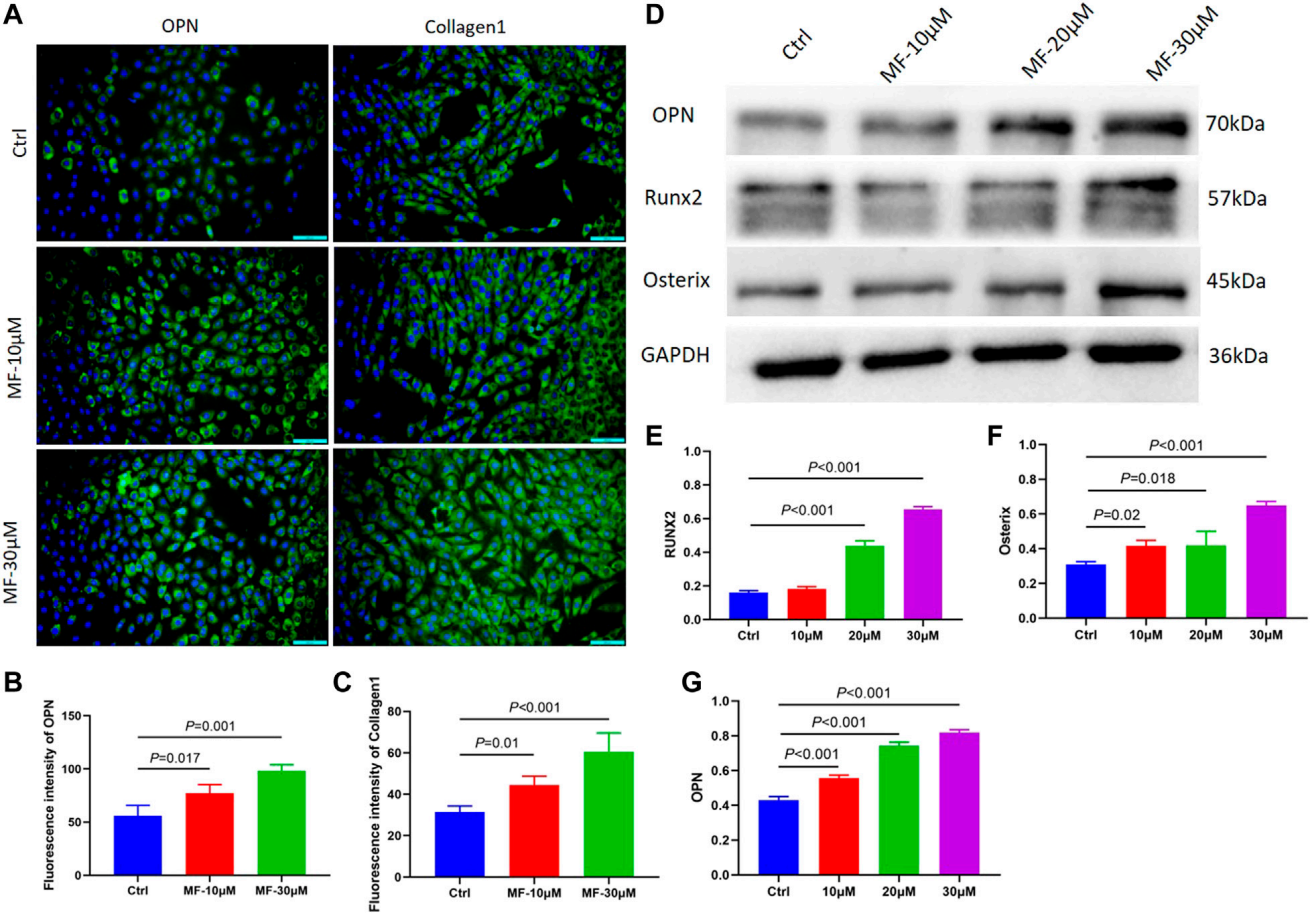
**(A–C)** The immunofluorescence of OPN and Collagen1 when the MC3T3-E1 cells were intervened by 10 μM and 30 μM mangiferin for 14 days. When compared with the control group, the OPN and Collagen1 significantly increased in the 10 μM and 30 μM (*p* < 0.05, respectively). **(D–G)** When compared with the control group, the protein level of OPN, Runx2, and Osterix significantly increased when the concentration was 10, 20, and 30 μM (*p* < 0.05, respectively). Note: MF, Mangiferin. Ctrl, osteogenic induction group.

To further explore whether the effect of Mangiferin was dose-dependent, the MC3T3-E1 cells were treated with 10 and 30 µM Mangiferin in an induced differentiation culture for 14 days. The mean immunofluorescence intensity of OPN and Collagen1 significantly increased (*p* < 0.05, Figures 2A–C).

The cells were treated with 10, 20, and 30 µM Mangiferin to further measure the protein expression of OPN, RUNX2, and Osterix. Compared to the control group, western blotting revealed a dose-dependent effect of Mangiferin on osteogenic differentiation, as the protein levels of OPN, RUNX2, and Osterix increased with the increasing concentrations of 10, 20, and 30 µM (*p* < 0.05) (Figures 2D–G).

### Mangiferin promoted cell proliferation and inhibited cell apoptosis of MC3T3-E1 cells

**FIGURE 3:**
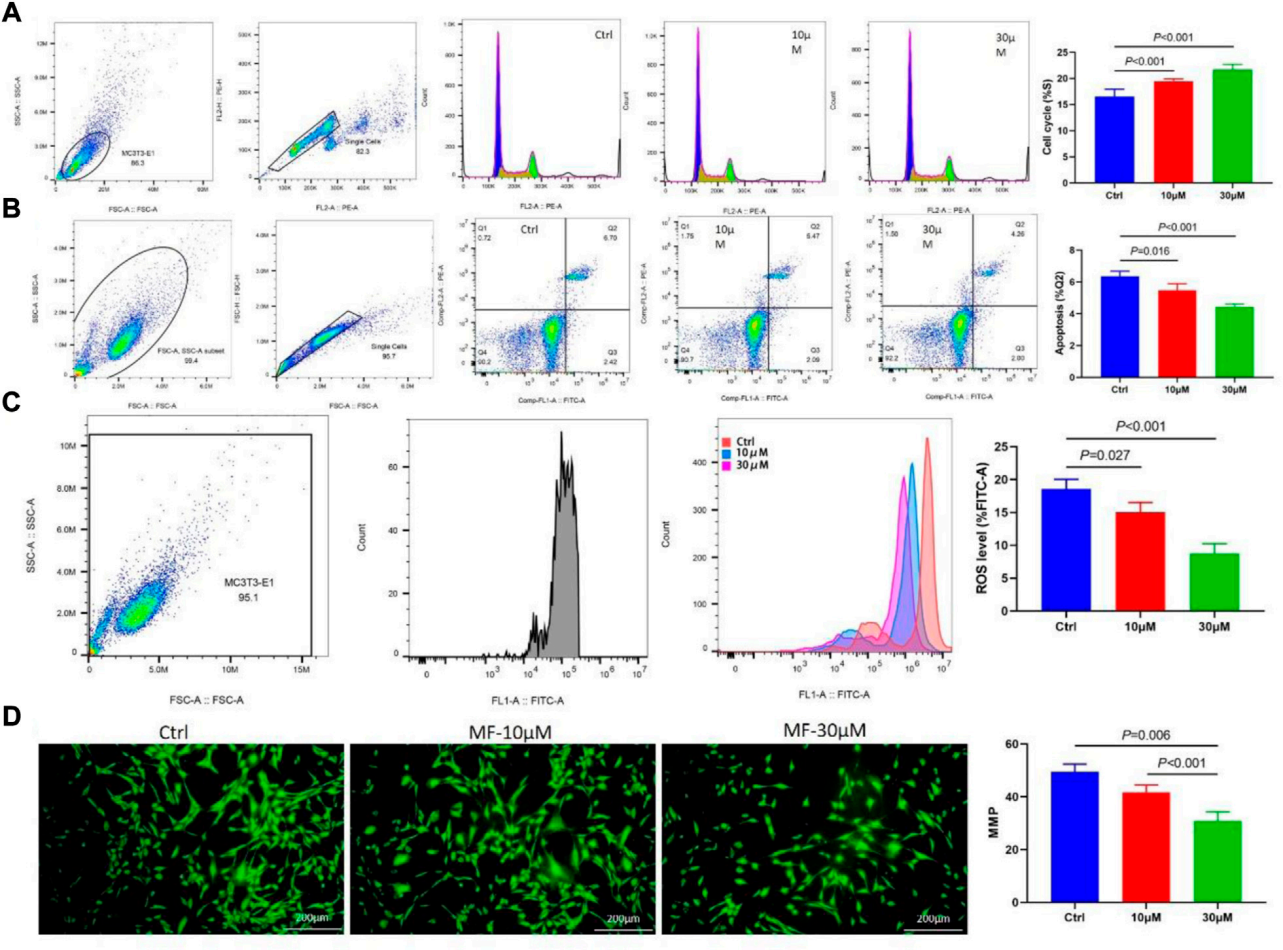
**(A)** Flow Cytometry of the cell cycle, when the concentrations were 10 μM and 30 μM, the percent of the S phase significantly increased when compared with the control group (*p* < 0.001, respectively). **(B)** Flow Cytometry of cell apoptosis in the Q2 phase significantly decreased when the concentrations were 10 μM and 30 μM (*p* < 0.05, respectively). **(C)** Flow Cytometry of ROS level significantly decreased when the concentrations were 10 μM and 30 μM (*p* < 0.05, respectively). **(D)** Immunofluorescence of mitochondrial membrane potential (MMP) significantly decreased when the concentrations were 10 μM and 30 μM (*p* < 0.01, respectively). Note: MF, Mangiferin. Ctrl, osteogenic induction group.

The cell cycle flow cytometry demonstrated that Mangiferin considerably increased the percentage of MC3T3-E1 cells in the S phase. The prolonged cell division was dose-dependent, and 30 µM of Mangiferin significantly promoted cell proliferation (*p* < 0.001, Figure 3A). Cellular apoptosis presented the opposite trend. Mangiferin at a concentration of 10 and 30 µM significantly inhibited the apoptosis of MC3T3-E1 cells (*p* < 0.05, *p* < 0.001, respectively, Figure 3B). The ROS level significantly decreased when the concentration was 10 and 30 µM (*p* < 0.05, Figure 3C). The immunofluorescence of MMP depicted that its destruction significantly decreased when treated with Mangiferin (*p* < 0.05, Figure 3D), a tendency comparable to cellular apoptosis.

### Mangiferin promoted osteogenic differentiation by targeting AXL/ERK5 pathway

**FIGURE 4:**
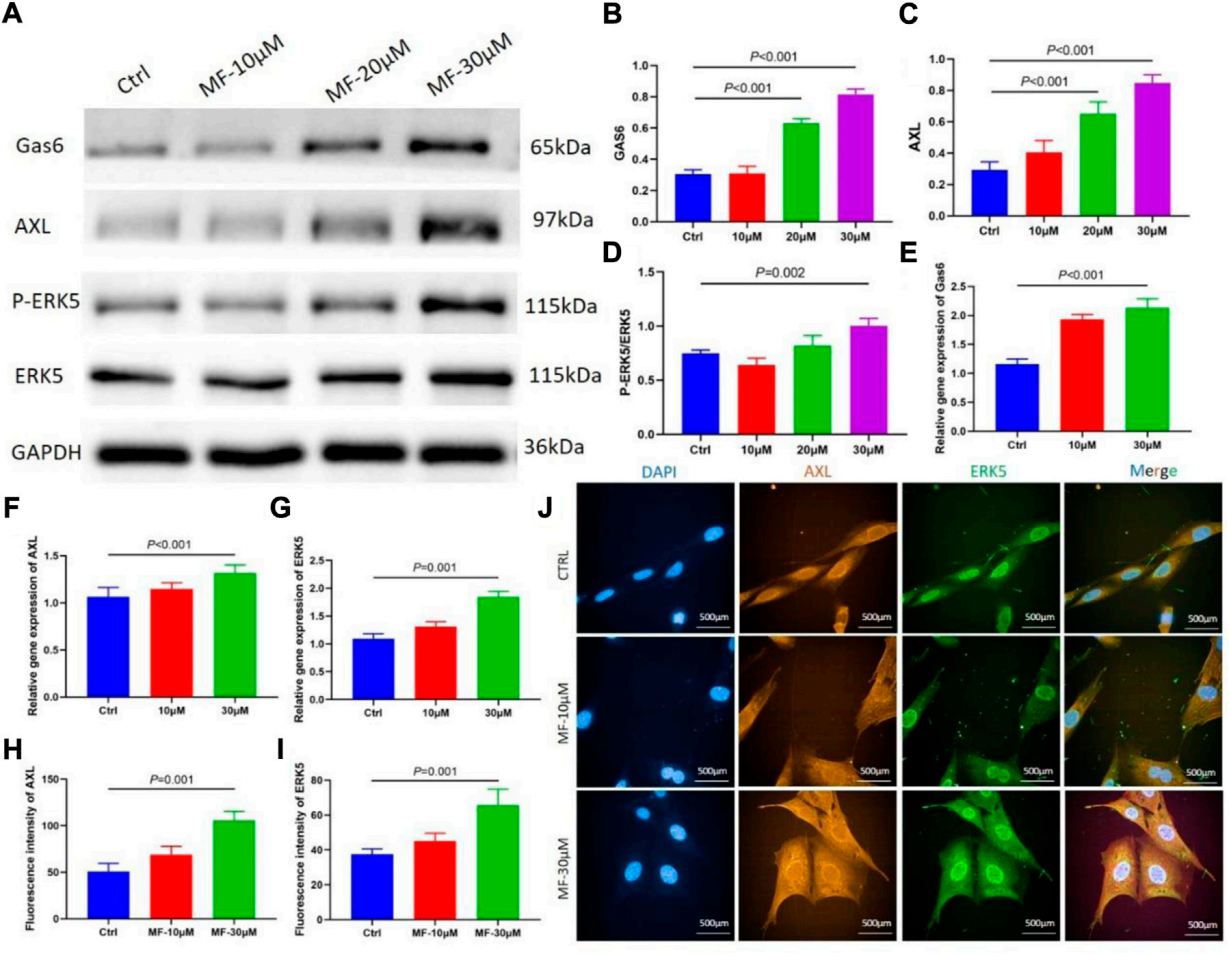
**(A–D)** When compared with the control group, the protein level of Gas6 and AXL significantly increased when the concentration was 10, 20, and 30 μM (*p* < 0.05, respectively). The ratio of P-ERK5/ERK5 significantly increased when the concentration was 30 μM (*p* < 0.01). **(E–G)** qRT-PCR showed that the relative gene expression of Gas6, AXL, and ERK5 significantly increased in the mangiferin group when the concentration was 30 μM (when compared with the control group, *p* < 0.01). **(H–J)** The immunofluorescence of AXL and ERK5 when the MC3T3-E1 cells were intervened by 10 μM and 30 μM mangiferin. When compared with the control group, the AXL and ERK5 significantly increased in the 30 μM (*p* < 0.01, respectively) Note: MF, Mangiferin. Ctrl, osteogenic induction group.

The protein level of P-ERK5 was evaluated by calculating the ratio of P-ERK5/ERK5, the western blotting results showed that the protein level of Gas6, AXL, and P-ERK5, and the results revealed that 30 µM Mangiferin significantly increased the ratio of P-ERK5/ERK5 (*p* < 0.05), the 20 µM and 30 µM concentration of mangiferin significantly promoted the protein level of Gas6 and AXL (*p* < 0.01, Figures 4A–D).

The qRT-PCR demonstrated that when the concentration of Mangiferin was 30 μM, the gene expression of Gas6, AXL, and ERK5 significantly increased compared to the control group (*p* < 0.01, Figures 4E–G).

The mean immunofluorescence intensity of AXL and ERK5 significantly increased when the MC3T3-E1 cells were intervented by a 30 µM concentration of Mangiferin (*p* < 0.05, Figures 4H–J).

### Knockdown of AXL inhibits the mangiferin-induced cell osteogenic differentiation of MC3T3-E1 cells

**FIGURE 5:**
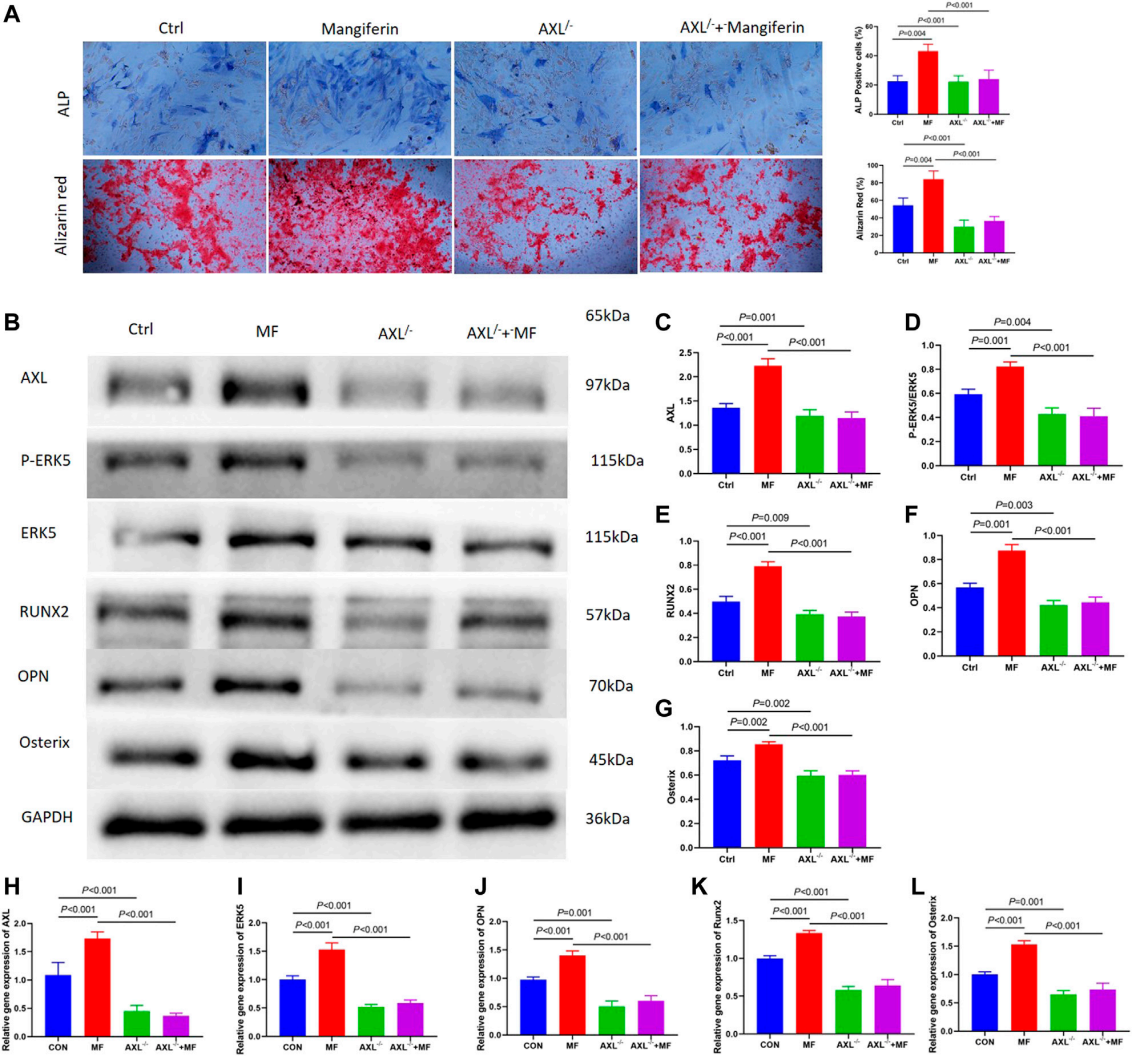
**(A)** Alkaline phosphatase (ALP) staining and alizarin red staining showed that Mangiferin significantly promotes osteogenic differentiation compared to the Ctrl group (*p* < 0.001). While it significantly decreased in the AXL^−/−^ group (*p* < 0.001). **(B–G)** Western blotting of AXL, P-ERK5, and ERK5, RUNX2, OPN, and Osterix in the control group, mangiferin group, AXL^−/−^group, and AXL^−/−^ + mangiferin group. The protein levels of AXL, P-ERK5/ERK5, RUNX2, OPN, and Osterix significantly increased in the mangiferin group (when compared with the control group, *p* < 0.01). While they significantly decreased in the AXL^−/−^ and AXL^−/−^ + mangiferin group (when compared with the control group and mangiferin group, *p* < 0.01, respectively). **(H–L)** qRT-PCR showed that the relative gene expression of AXL, ERK5, OPN, Osterix, and Runx2 significantly increased in the mangiferin group (when compared with the control group, *p* < 0.01). While they significantly decreased in the AXL^−/−^ and AXL^−/−^ + mangiferin group (when compared with the control group and mangiferin group, *p* < 0.001, respectively). Note: MF, mangiferin, Ctrl, osteogenic induction group.

Alkaline phosphatase (ALP) staining and alizarin red staining showed that Mangiferin significantly promotes osteogenic differentiation compared to the Ctrl group (*p* < 0.001). While it significantly decreased in the AXL^−/−^ group (*p* < 0.001) (Figure 5A). The protein and gene levels of AXL significantly decreased in the AXL^−/−^ group relative to the control group (*p* < 0.01, Figures 5A,B,G), indicating that AXL was successfully knocked down. Compared to the control group, the levels of P-ERK5, RUNX2, OPN, and Osterix increased significantly (*p* < 0.001) in the Mangiferin intervented group, while they decreased significantly (*p* < 0.001) in the AXL^−/−^ + Mangiferin group (Figures 5B–G).

qRT-PCR revealed that the relative gene expression of AXL, ERK5, OPN, Osterix, and RUNX2 increased significantly in the Mangiferin group (compared to the control group, *p* < 0.01). While they decreased significantly in the AXL^−/−^ and AXL^−/−^ + Mangiferin group (compared to the control group and Mangiferin group, *p* < 0.001, Figures 5H–L). There was no significant difference in the protein and gene levels of P-ERK5 (ERK5), OPN, Osterix, and RUNX2 between the AXL^−/−^ and AXL^−/−^ + Mangiferin group, indicating that it may promote osteogenic differentiation by targeting AXL/ERK5 pathway.

### Mangiferin allievate osteoporosis of the ovariectomized mouse

**FIGURE 6:**
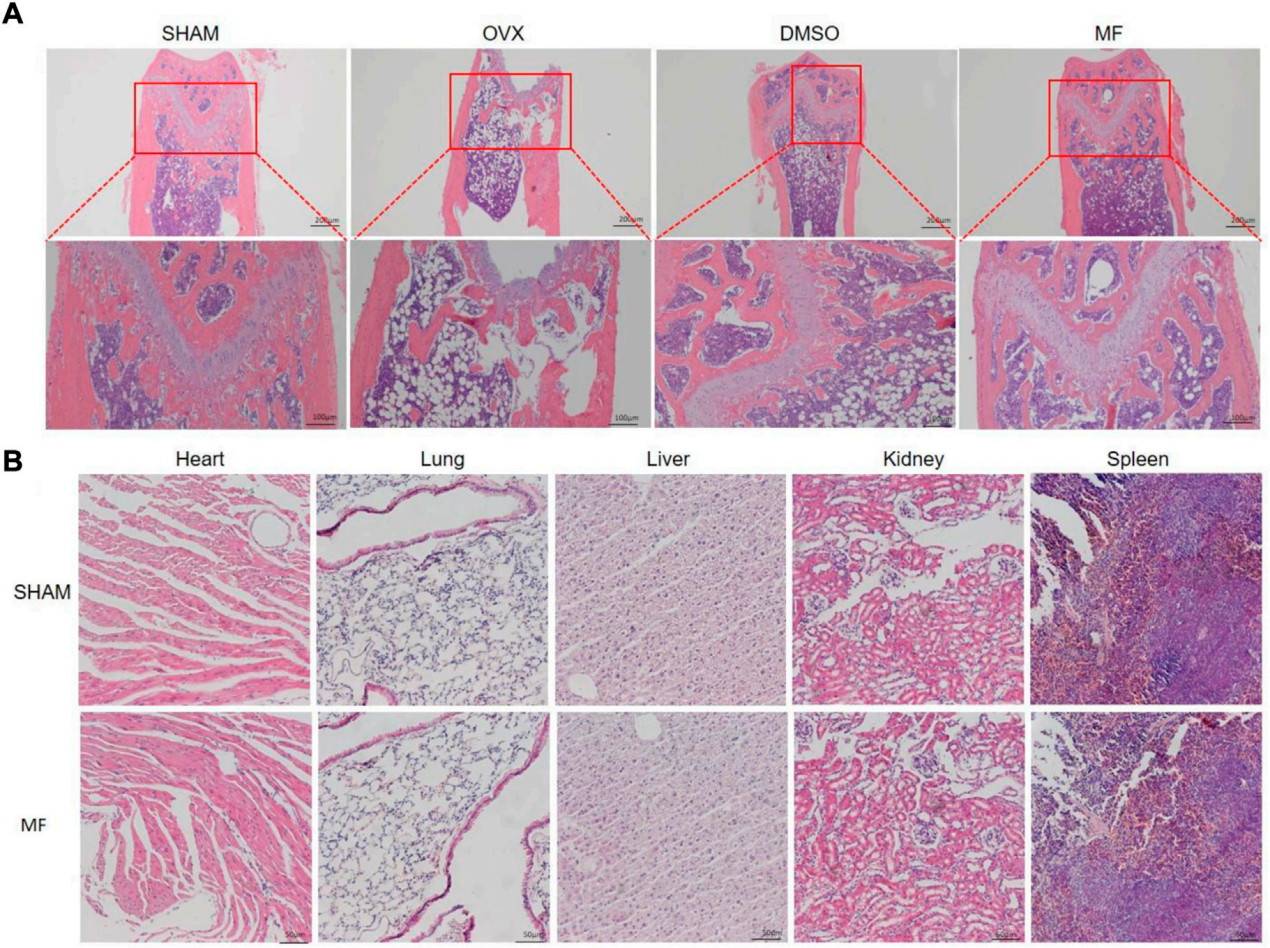
H and E staining of the femur of OVX mice. **(A)** trabecular bone was ruptured and scattered in the OVX and DMSO groups, while the pathological changes in the mangiferin group were less severe than that of the OVX group. **(B)** H and E staining of the heart, lung, liver, kidney, and spleen showed that the mangiferin has no obvious organ toxicity. Note: MF, Mangiferin.

The H and E staining of the femur demonstrated that ovariectomy successfully induced osteoporosis. Compared to the SHAM group, the trabecular bone was thin and scattered, and the number of trabecular decreased; the trabecular gap was widened in the OVX and DMSO groups, while the osteoporosis was less severe in the Mangiferin group (Figure 6A). H and E staining revealed that the morphology of the heart, liver, spleen, lung, and kidneys in the Mangiferin group was normal and not significantly different from that of the control group, suggesting that the intraperitoneal injection of 40 mg/kg/d Mangiferin did not cause apparent organ toxicity in mice (Figure 6B).

**FIGURE 7:**
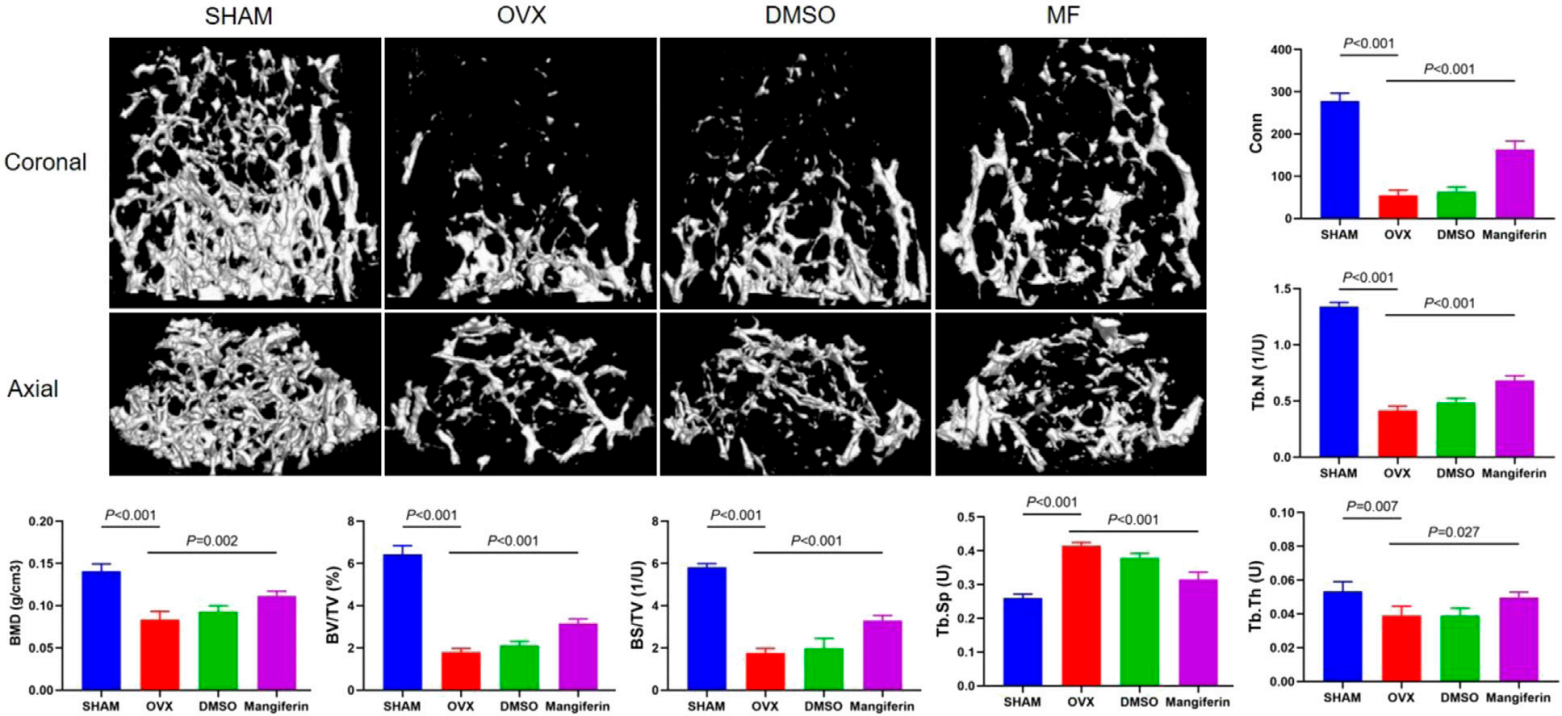
Micro-CT of the femur in the SHAM, OVX, DMSO, and mangiferin group. When compared with the SHAM group, the value of BMD, BV/TV, BS/TV, Tb. Th, Tb. N, and Conn significantly decreased in the OVX and DMSO groups (*p* < 0.01, respectively). When compared with the OVX group, the level of BMD, BV/TV, BS/TV, Tb. Th, Tb. N, and Conn significantly increased in the mangiferin group (*p* < 0.05, respectively). While the value of Tb. Sp presented the opposite trend. Note**:** MF, Mangiferin.

Meanwhile, the micro-CT revealed that osteoporosis was most severe in the OVX and DMSO groups, whereas it was least severe in the Mangiferin group. Compared to the SHAM group, BMD, BV/TV, BS/TV, Tb. Th, Tb. N, and Conn that were related to the severity of osteoporosis increased significantly in the OVX and DMSO groups (*p* < 0.01). Compared to the OVX group, these levels decreased significantly in the Mangiferin group (*p* < 0.05) except for Tb. Sp, which presented an opposite trend (Figure 7).

### Mangiferin promotes osteogenic differentiation in the ovariectomized mouse

**FIGURE 8:**
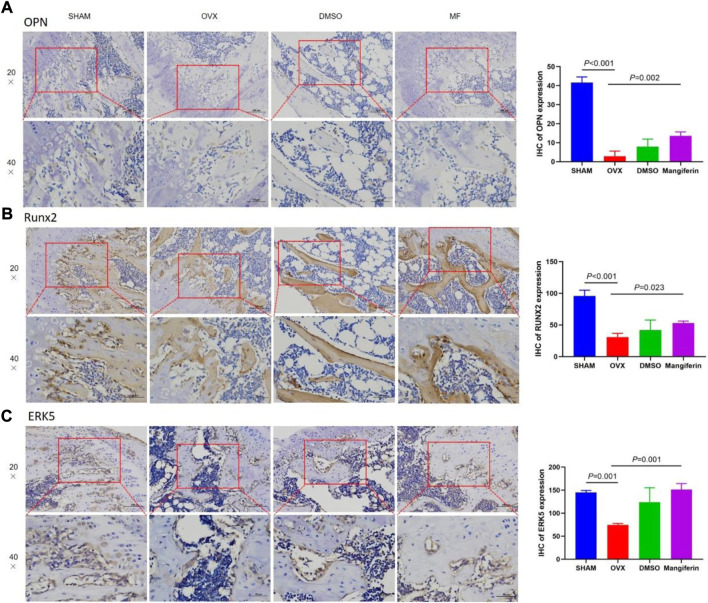
IHC of the femur, in the SHAM, OVX, DMSO, and mangiferin group. The levels of OPN **(A)**, RUNX2 **(B)**, and ERK5 **(C)** the OVX group significantly decreased when compared with the SHAM group (*p* < 0.01, respectively), while they significantly increased in the mangiferin group when compared with the OVX group (*p* < 0.05, respectively).

The IHC staining depicted that the expression of OPN and RUNX2 decreased significantly in the OVX and DMSO groups (as compared to the SHAM group, *p* < 0.05), but Mangiferin reversed this trend (compared to the OVX group, *p* < 0.05, Figures 8A,B).

### Mangiferin promotes osteogenic differentiation in the ovariectomized mouse by targeting AXL/ERK5 pathway

**FIGURE 9:**
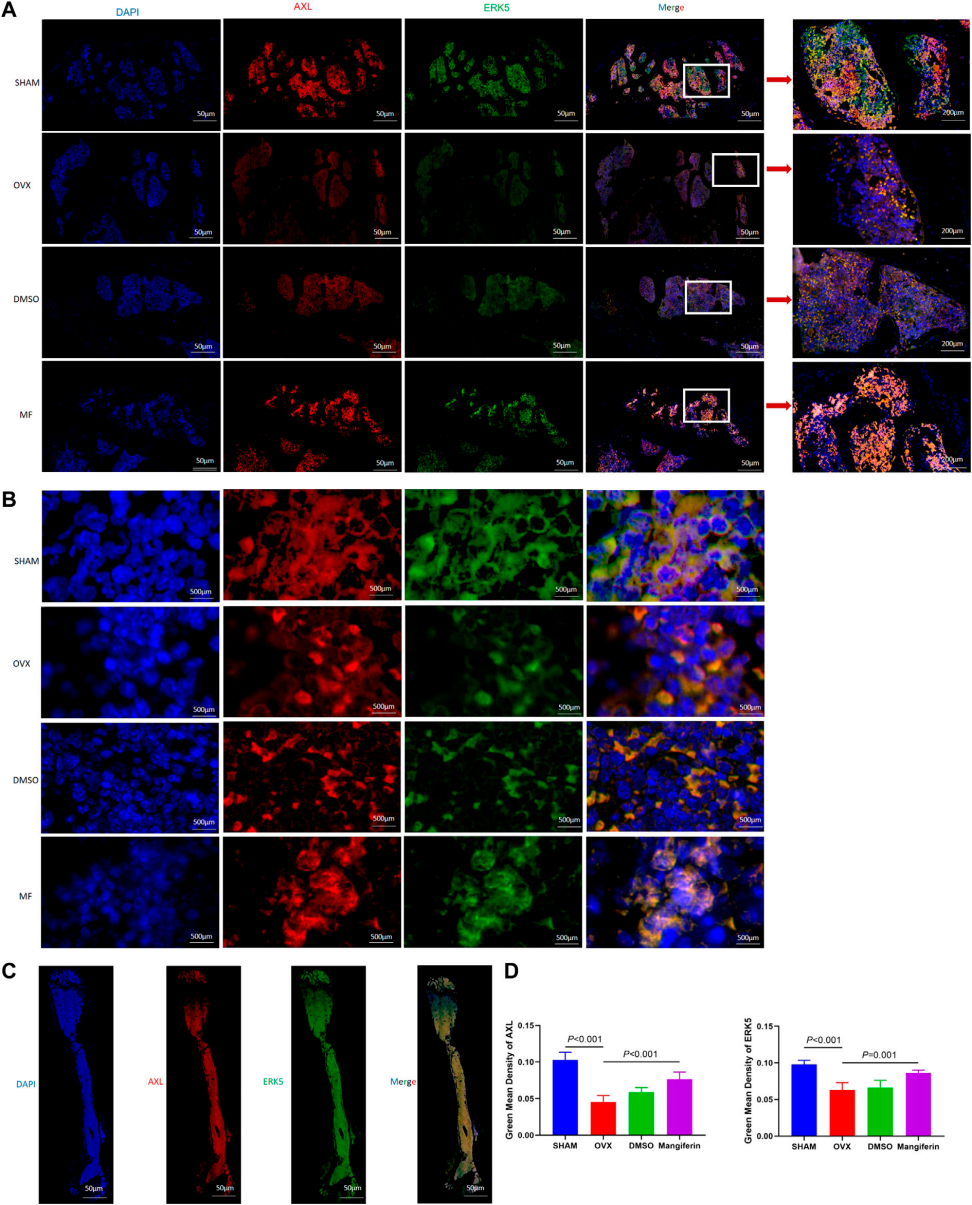
Immunofluorescence of the AXL and ERK5 in the femur. **(A)** Local view of AXL and ERK5 expression in the femoral metaphysis. **(B)** Localization of expression of AXL and ERK5 in the femoral metaphysis, amplificated images. **(C)** Panoramic view of the femur, DAPI (blue), ERK5 (green), AXL (red), and merged version. **(D)** The green mean density of AXL and ERK5 in the OVX and DMSO groups significantly decreased when compared with the SHAM group (*p* < 0.001, respectively). The green mean density of AXL and ERK5 significantly increased in the mangiferin group when compared with the OVX and DMSO groups (*p* < 0.05, respectively) Note: MF, Mangiferin.

The IHC staining depicted that the expression of ERK5 decreased significantly in the OVX and DMSO groups (as compared to the SHAM group, *p* < 0.05), but Mangiferin reversed this trend (compared to the OVX group, *p* < 0.05, Figure 8C). Immunofluorescence colocalization of the femur demonstrated that ERK5 was expressed in the cytoplasm and nucleus, and AXL was mainly expressed in the cytoplasm (Figures 9A–C). Compared to the SHAM group, the fluorescence intensity of AXL and ERK5 significantly decreased in the OVX and DMSO groups (*p* < 0.05). While Mangiferin intervention increased the expression level of AXL and ERK5 relative to the OVX and DMSO groups (*p* < 0.01, Figure 9D).

**FIGURE 10:**
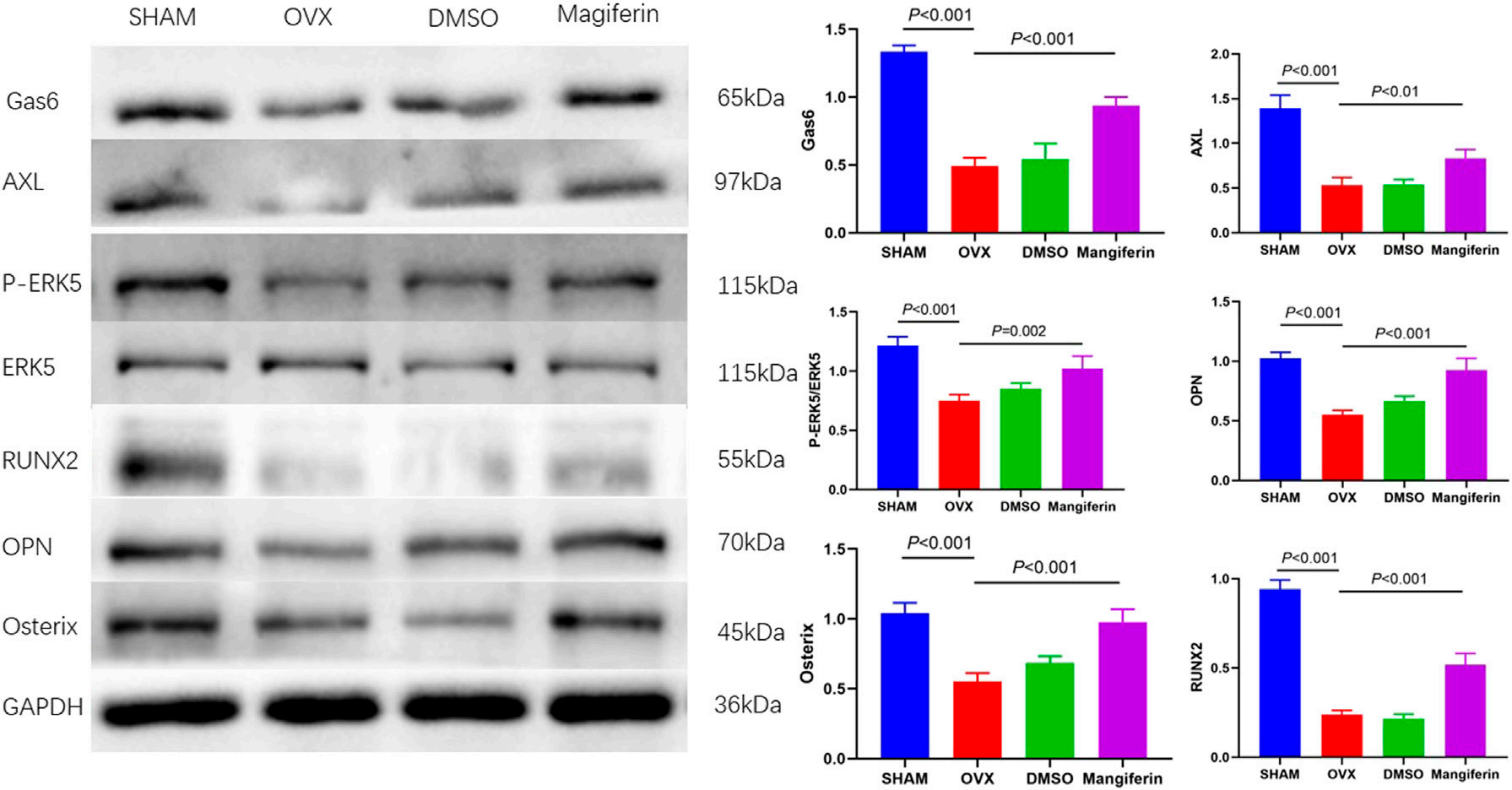
Western blot of the femur in the SHAM, OVX, DMSO, and mangiferin group. The protein levels of Gas6, AXL, P-ERK5/ERK5, OPN, Osterix, and RUNX2 significantly decreased in the OVX and DMSO group (when compared with the SHAM group, *p* < 0.001, respectively), while they significantly increased in the mangiferin group (when compared with the OVX and DMSO groups, *p* < 0.05, respectively).

Western blotting of bone tissue revealed that the levels of Gas6, AXL, P-ERK5, RUNX2, OPN, and Osterix significantly decreased in the OVX and DMSO groups relative to the SHAM group (*p* < 0.001), but Mangiferin reversed the trend (*p* < 0.01) (Figure 10).

## Discussion

**FIGURE 11:**
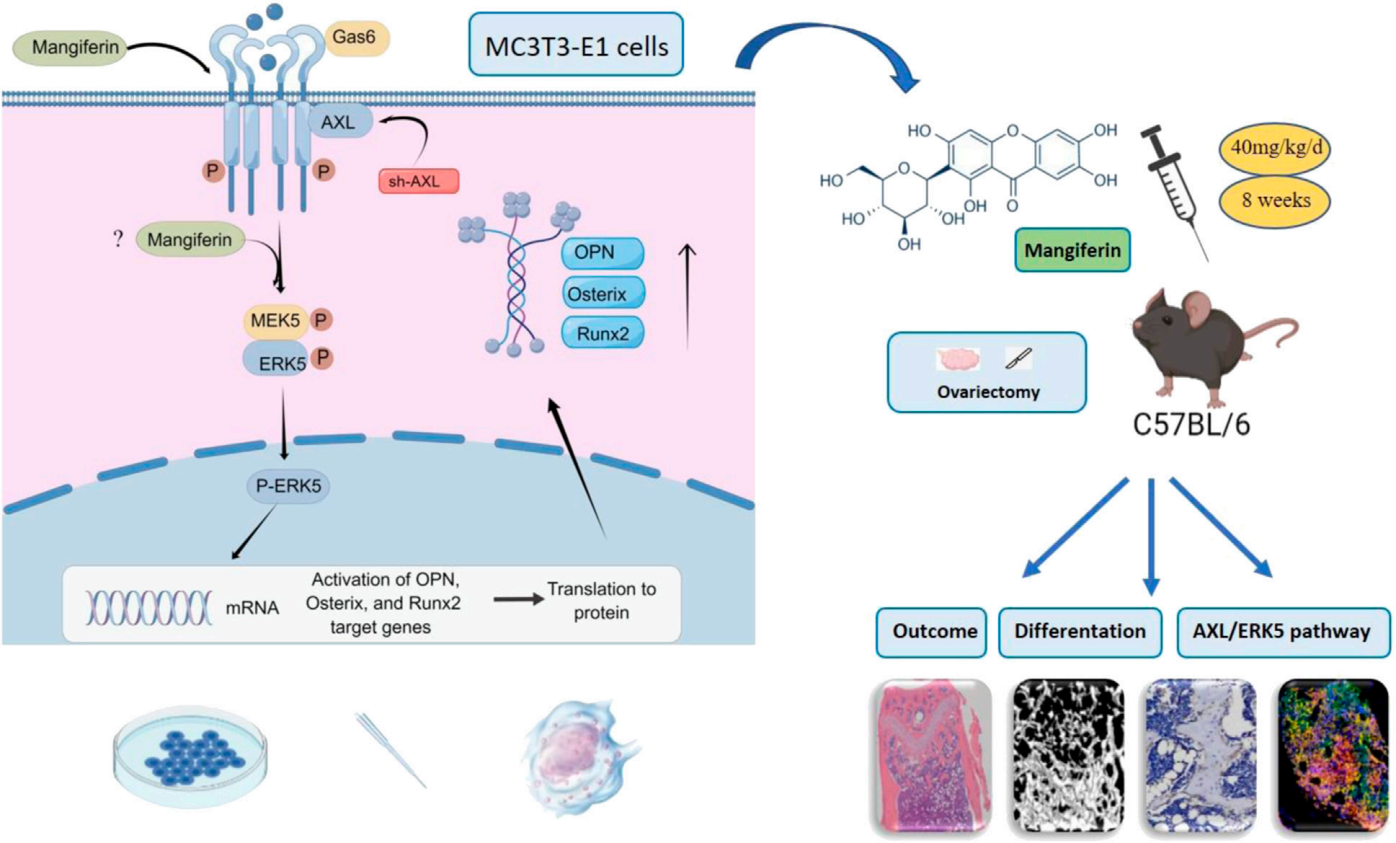
Mangiferin promoted osteogenic differentiation of MC3T3-E1 cells and alleviated osteoporosis in OVX mice. The potential mechanism was *via* the AXL/ERK5 pathway.

This study revealed that Mangiferin promotes osteogenic differentiation of MC3T3-E1 cells and alleviates osteoporosis in OVX mice, with AXL/ERK5 as a potential pathway (Figure 11). Mangiferin promoted osteogenic differentiation dose-dependently when the concentration was less than 30 μM, and at 30 μM, it significantly upregulated the AXL and ERK5 levels.

Mangiferin is a glucosylxanthone extracted from the stem bark of Mangifera (Rodeiro et al., 2012). The chemical name of Mangiferin is 2-β-d-glucopyranosyl-1,3,6,7-tetrahydroxyxanthone (Muruganandan et al., 2002), also called C-glucosyl xanthone (Scartezzini and Speroni, 2000). In published investigations, the purity of Mangiferin isolated from the bark, fruits, roots, and leaves of Mangifera indica Linn was approximately 95.5% (Muruganandan et al., 2002; Prabhu et al., 2006). The purity of the purified Mangiferin can be greater than 98%. We used 98.11% purified Mangiferin procured from Selleck Chemical.

In the early research, it was found that Mangiferin has a therapeutic effect on periodontal disease; the alveolar bone loss was significantly inhibited by suppressing the inflammatory activity (Duang et al., 2011) and anti-chemotaxis action (Carvalho et al., 2009). Li et al. (2017) demonstrated that PLGA scaffolds loaded with Mangiferin could repair the alveolar bone and attenuate the decrease of cell viability in diabetic patients. Based on its antioxidative and anti-inflammatory effect, Mangiferin has been identified as a potential treatment option for rheumatoid arthritis (Luczkiewicz et al., 2014).

In addition, Mangiferin is an effective ingredient in traditional Chinese medicine like Er-Xian Decoction and Tsu-kan-gan (Li et al., 1998; Qin et al., 2008), that can reduce bone resorption. Mangiferin-loaded biomaterials, such as chitosan-silica hybrid scaffolds, were proposed to be beneficial for bone regeneration (Demeyer et al., 2021). Ding et al. found that Mangiferin inhibits oxidative stress and apoptosis in MC3T3-E1 cells *via* targeting the BMP2/Smad-1 pathway (Ding et al., 2018). Ang et al. found that Mangiferin can inhibit osteogenesis and bone resorption by targeting the NF-κB and ERK pathways (Ang et al., 2011). Bai et al., 2018 demonstrated that Mangiferin promotes ossification-based bone repair by regulating endochondral ossification; inducing autophagy was the potential mechanism. Furthermore, Mangiferin can alleviate the inhibition of chondrogenic differentiation of mesenchymal stem cells, thereby promoting cartilage repair (Huh et al., 2014). Although it was found that Mangiferin and its derivatives inhibit bone absorption, very few studies have been conducted on its pharmacology. Most studies focused on osteoclast function, but the effect on osteogenic differentiation still needs to be explored.

According to our previous studies, ERK5 is essential for the differentiation and proliferation of MC3T3-E1 cells, making it a suitable target for the treatment of osteoporosis. Mitogen-activated protein kinase (MAPK) signaling is a classical pathway that regulates cellular events, including proliferation, apoptosis, differentiation, and cell migration (Lu and Malemud, 2019). An extracellular signal-regulated kinase (ERK) is a member of the MAPK family and has been demonstrated to play an important role in regulating cellular events (Cargnello and Roux, 2011). Yan et al. (2022) found that Mangiferin Alleviates Postpartum Depression-Like Behaviors by Inhibiting MAPK pathway. Thus we speculate that the MAPK signaling is an important target of Mangiferin.

AXL is one of the TAM (Tyro3/Axl/Mer) receptor families, the intracellular segment of AXL has kinase activity and can participate in the transmission of various signaling pathways in normal cells and tumor cells. In the research on Alzheimer’s disease (AD), it was found that the AXL/ERK signaling pathway is involved in the proliferation and phenotypic transformation of microglia. Up-regulation of molecules related to this signaling pathway can improve the cognitive deficit of AD (Zhang et al., 2018). In addition, It was found that AXL regulates the proliferation, cycle, migration, and differentiation of osteoblasts and osteoclasts in osteosarcoma (Gobin et al., 2014). Therefore, the AXL/ERK signaling pathway can be a promising mechanism in the regulation of proliferation and differentiation of various cells. The present study revealed that Mangiferin can promote osteogenic differentiation *via* AXL/ERK5 pathway.

We found that Mangiferin was dose-dependent in promoting osteogenic differentiation at concentrations below 30 μM, and 30 µM Mangiferin was essential for upregulating the expression of AXL and ERK5. Ding et al. used 30, 40, and 60 µM of Mangiferin in dexamethasone-induced MC3T3-E1 cells. They found that the effect was dose-dependent, with 60 µM Mangiferin considerably reversing the inhibition of cell viability induced by dexamethasone (Ding et al., 2018). Ang et al. found that 0.5 and 1 mM Mangiferin significantly inhibits bone resorption in osteoclastogenesis (Ang et al., 2011). Sekiguchi et al. utilized a similar concentration (Sekiguchi et al., 2017). Huh et al. reported that Mangiferin at 1, 10, and 20 µM can enhance chondrogenic differentiation of mesenchymal stem cells in a dose-dependent manner. Bai et al. (2018) revealed that Mangiferin did not cause cell toxicity at concentrations less than 100 µM and that hypoxia-induced cellular apoptosis was prevented at concentrations more than 20 µM. Li et al. (2019b) found that a lower than 200 µM of Mangiferin does not inhibit a chondrocyte’s viability. Consistent with our findings, most investigations found that the effect was dose-dependent. In most studies, the duration of induced differentiation of MC3T3-E1 cells was from seven to 21 days (Wang et al., 2019; Yang et al., 2019; Jeon et al., 2021). We demonstrated that 14-day induction and intervention with Mangiferin significantly promoted osteogenic differentiation of MC3T3-E1 cells.

The osteogenic activity of Mangiferin was further verified *in vivo*. For the animal model of osteoporosis, OVX mice are commonly employed to imitate postmenopausal osteoporosis. The lethal dose of 50 (LD50) of Mangiferin administered orally was 400 mg/kg (Jagetia and Baliga, 2005). We used a 40 mg/kg dose of Mangiferin through the gavage, following the instructions and dosage in prior reports (Huang et al., 2020). We found that the 40 mg/kg Mangiferin had no obvious organ toxicity, activated AXL/ERK5 pathway, and promoted osteogenic differentiation in OVX mice. H and E, IHC, and micro-CT identified the treatment effect of Mangiferin. The immunofluorescence of femur bone showed that AXL/ERK5 pathway is expressed in the cytoplasm and is concentrated in the bone marrow and metaphysis. We speculated that the alleviation of osteoporosis is partially attributed to AXL/ERK5 pathway upregulation and the stimulation of osteogenic differentiation.

This study had some limitations. First, the effect of Mangiferin on osteoporosis was based on osteogenic differentiation. Since osteoporosis is a comprehensive pathological change, the proliferation, apoptosis of osteoblast, and biological behavior of osteoclast were not investigated. Second, the mechanism was not verified in multiple cells, such as fetal osteoblasts and BMSCs. Third, ovariectomy-induced osteoporosis is one of the most commonly used animal models in experimental research, and it simulates postmenopausal osteoporosis in older women. However, as osteoporosis is a systemic disease, the disuse osteoporosis and pathological osteoporosis were not included in this study. Furthermore, the clinical applicability of insights drawn from rodent models is restricted. Future research should focus on more specific mechanisms and comprehensive models, including BMSCs, primary cells, and the interaction of proteins.

## Data Availability

The original contributions presented in the study are included in the article/supplementary material, further inquiries can be directed to the corresponding authors.

## Glossary

CCK-8: Cell count kitting-8
GAS6: Growth arrest specific 6
ERK5: Extracellular regulated protein kinases 5
BMP2: Bone morphogenetic protein-2
ALP: Alkaline phosphatase
OVX: Ovariectomized
PBS: Phosphate buffer saline
EDTA: Ethylene diamine tetra acetic acid
NF-Κb: Nuclear factor-k-gene binding
Nrf2: Nuclear factor erythroid-2
MEM: Minimum essential medium
SPF: Specific pathogen free
DMSO: Dimethyl sulfoxide
PEG: PolyEthylene Glycol
IHC: Immunohistochemistry
BMD: Bone mineral density
BV: Bone volume
BS: Bone surface
TV: Total volume
BV/TV: Bone volume fraction
BS/TV: Bone surface density
Tb. Sp: Trabecular Separation/Spacing
Tb. Th: Trabecular Thickness
Tb. N: Trabecular number
Conn: Connectivity
RIPA: Radio-Immunoprecipitation Assay
PMSF: Phenylmethanesulfonyl fluoride
BCA: Bicinchonininc acid
SDS-PAGE: Sodium dodecyl sulfate-polyacrylamide gel electrophoresis
PVDF: Polyvinylidene fluoride
GAPDH: Glyceraldehyde-3-phosphate dehydrogenase
ECL: Enhanced Chemiluminescence
DAPI: 4,6-diamino-2-phenyl indole
IOD: Integrated optical density
ROS: Reactive oxygen species
ANOVA: One-way Analysis of Variance
BMSCs: Bone marrow mesenchymal stem cells
LD50: Lethal dose 50
